# A short peptide LINC00665_18aa encoded by lncRNA LINC00665 suppresses the proliferation and migration of osteosarcoma cells through the regulation of the CREB1/RPS6KA3 interaction

**DOI:** 10.1371/journal.pone.0286422

**Published:** 2023-06-07

**Authors:** Junwei Pan, Ming Liu, Xiaozong Duan, Dan Wang

**Affiliations:** Department of Orthopedics, The First Affiliated Hospital of Zhengzhou University, Zhengzhou, China; Universita degli Studi della Campania Luigi Vanvitelli, ITALY

## Abstract

Long noncoding RNAs (lncRNAs) encompass short open reading frames (sORFs) that can be translated into small peptides. Here, we investigated the encoding potential of lncRNA LINC00665 in osteosarcoma (OS) cells. Bioinformatic analyses were utilized to predict the lncRNAs with encoding potential in human U2OS cells. Protein expression was assessed by an immunoblotting or immunofluorescence method. Cell viability was assessed by cell counting Kit-8 (CCK-8). Cell proliferation was detected by 5-ethynyl-2’-deoxyuridine (EdU) assay. Cell migration was gauged by transwell assay. The downstream effectors of the short peptide were verified using qualitative proteome analysis after immunoprecipitation (IP) experiments. The effect of the short peptide on protein interactions were confirmed by Co-Immunoprecipitation (CoIP) assays. We found that lncRNA LINC00665 encoded an 18-amino acid (aa)-long short peptide (named LINC00665_18aa). LINC00665_18aa suppressed the viability, proliferation, and migration of human MNNG-HOS and U2OS OS cells *in vitro* and diminished tumor growth *in vivo*. Mechanistically, LINC00665_18aa impaired the transcriptional activity, nuclear localization, and phosphorylation of cAMP response element-binding protein 1 (CREB1). Moreover, LINC00665_18aa weakened the interaction between CREB1 and ribosomal protein S6 kinase A3 (RPS6KA3, RSK2). Additionally, increased expression of CREB1 reversed the inhibitory effects of LINC00665_18aa on OS cell proliferation and migration. Our findings show that the short peptide LINC00665_18aa exerts a tumor-inhibitory function in OS, providing a new basis for cancer therapeutics through the functions of the short peptides encoded by lncRNAs.

## Introduction

Despite a rare cancer, osteosarcoma (OS) remains the most prevalent malignancy of the bone occurred in children and adolescents [1]. OS is thought to derive from bone-forming mesenchymal cells [2]. The 5-year survival rate of OS with localized tumors is about 70%, while patients with metastatic OS have poor survival outcomes with 5-year survival rate of less than 30% [3]. The typically combined regimen of surgery and chemotherapeutic agents can control numerous primary OS tumors effectively, but these therapies have limited effects in reining the metastatic progression and recurrence after treatment [1, 4]. Molecularly targeted treatment and immunotherapy targeting overexpressed proteins are in active clinical development for OS [5]. Robust preclinical models and patient-derived organoids are under intensive exploration for OS at present [5, 6]. Therefore, more innovative, and effective treatment options are needed to improve the efficiency of OS therapy.

As a heterogeneous type of noncoding RNAs (ncRNAs), long ncRNAs (lncRNAs) exert critical functions in various biological processes implicated in human diseases [7]. Particularly in cancer, lncRNAs have established vital roles as tumor suppressors or oncogenic promoters in almost all types of cancer [8]. Although lncRNAs are initially defined as RNA transcripts without protein-coding potential, the development of the profiling of genome translation and ribosome has uncovered that some lncRNAs harbor short open reading frames (sORFs) that can interact with ribosomes and can be translated into small peptides [9, 10]. Recent documents have demonstrated the critical implication of lncRNA-encoded short peptides in cancer biology [9]. LINC00665, an annotated lncRNA transcript, has been identified as a tumor driver in various cancers [11]. In OS, LINC00665 operates as a sponge of certain miRNAs and thus enhances cancer cell growth and metastasis [12, 13]. Intriguingly, recent reports have documented that lncRNA LINC00665 encodes a 52-amino acid (aa)-long short peptide CIP2A-BP in triple-negative breast cancer and hepatocellular carcinoma, where the short peptide is capable of participating in cancer pathogenesis [14, 15]. However, the encoding potential of lncRNA LINC00665 in OS has not yet been explored.

Aberrant activation of cAMP response element-binding protein 1 (CREB1), a crucial transcriptional factor, has been reported to drive tumor progression in various cancers [16, 17], including OS [18, 19]. Ribosomal protein S6 kinase A3 (RPS6KA3, RSK2) is a downstream substrate of the ERK pathway and possesses a crucial function in the pathogenesis of OS [20, 21]. Importantly, RSK2 has been identified to phosphorylate and activate CREB1 [22], and the RSK2/CREB pathway plays an essential role in OS progression [23].

Here, focusing on the encoding potential of lncRNA LINC00665, we first demonstrated that lncRNA LINC00665 can encode an 18-aa-long short peptide (named LINC00665_18aa). Consequently, we further investigated the biological effect and mechanism of LINC00665_18aa on OS cell proliferation and migration.

## Materials and methods

### Bioinformatic analysis

To obtain the lncRNAs associated with ribosomes (count > 0) in OS cells, we utilized the Ribosome sequencing (Ribo-Seq) dataset of U2OS cells from the Translatome database-Species (http://www.translatomedb.net/searchspecies.html?sp=Human). The lncRNA-encoded peptides matched with human proteome were eliminated using UniProt database (https://www.uniprot.org/). The lncRNAs with encoding potential were retrieved from FuncPEP database (https://bioinformatics.mdanderson.org/Supplements/FuncPEP/database.html). Using DAVID database (https://david.ncifcrf.gov/home.jsp), we conducted Gene Ontology (GO) and Kyoto Encyclopedia of Genes and Genomes (KEGG) enrichment analyses. The information of all human transcriptional factors was downloaded from JASPAR database (https://jaspar.genereg.net/). To analyze protein interaction networks, we used String database (https://cn.string-db.org/). To observe the interaction relationships between CREB1 and other proteins, we utilized Genemania online tool (http://genemania.org/).

### Cell lines

Human MNNG-HOS and U2OS OS cell lines were procured from Procell (Wuhan, China). Cells were cultivated in Dulbecco’s modified Eagle medium (DMEM, Procell) medium in the presence of fetal bovine serum (10% v/v, Procell) and penicillin/streptomycin (1%, Procell) in a 5% CO_2_ atmosphere at 37°C.

### Plasmid and lentivirus constructs

**Fig 1:**
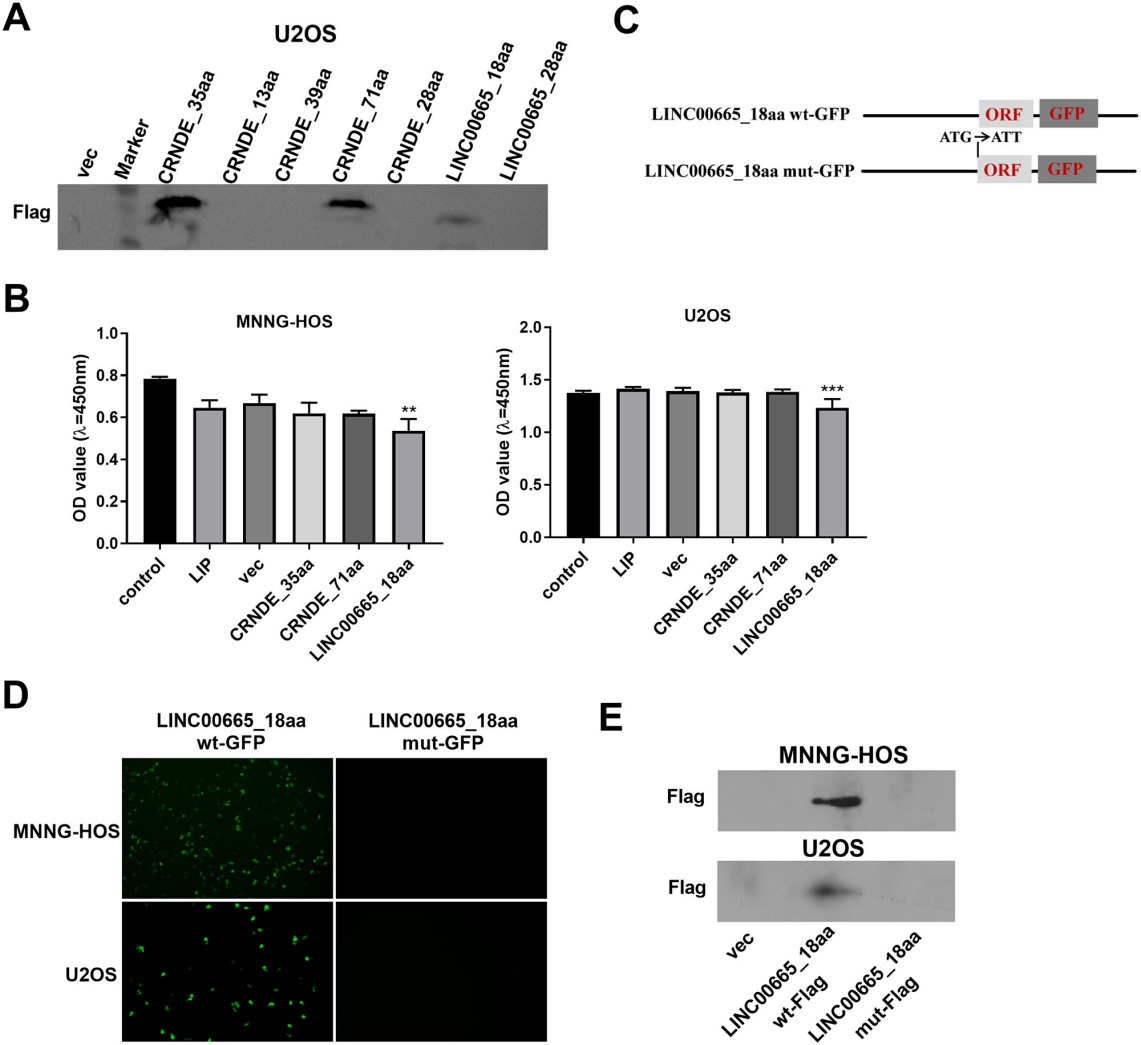
The lncRNA LINC00665 encodes a short peptide. (A) Immunoblotting analysis of the short peptides encoded by the indicated seven sORFs in U2OS cells after transfection by the corresponding expression constructs. (B) CCK-8 assay showing the effects of three short peptides on cell viability. (C) Diagram of the GFP fusion plasmids. (D) Representative GFP fluorescence images depicting the expression of the LINC00665_18aa-GFP fusion protein in GFP fusion plasmids-transfected cells. (E) Immunoblots of the LINC00665_18aa-Flag fusion protein in Flag fusion constructs-transfected cells. **P<0.01, ***P<0.001.

The following expression constructs (Genecreate, Wuhan, China) were used: pcDNA3.1-Flag tag vector-based expression plasmids encompassing the seven sORFs (shown in Fig 1A) and negative control vec, LINC00665_18aa expression plasmid fused with green fluorescent protein (GFP) tag (LINC00665_18aa wt-GFP) and its mutation (LINC00665_18aa mut-GFP, in which the start codon ATG of the LINC00665_18aa ORF was mutated to ATT), and the mutant-type LINC00665_18aa expression plasmid fused with Flag tag (LINC00665_18aa mut-Flag, in which the start codon ATG of the LINC00665_18aa ORF was mutated to ATT). The CREB1-Luc luciferase reporter plasmid was based on the pGL3 vector and obtained from Genecreate. The pRL-TK *Renilla* control plasmid was purchased from Miaoling Bio (Wuhan, China). The lentiviral particles expressing LINC00665_18aa and control particles were purchased from Genecreate.

### Transient transfection and lentivirus transduction

For plasmid transfection, the RFect Plasmid DNA Transfection Reagent was used as per the manufacturing protocols (Baidai, Changzhou, China). Briefly, a mix of 1 μg plasmid construct and 4 μL transfection reagent was prepared and then added into 1 × 10^5^ MNNG-HOS or U2OS cells. The transfected cells were harvested 24 h post-transfection for the subsequent assays.

For lentivirus transduction, 1 × 10^6^ U2OS cells were infected with lentiviral particles at a multiplicity of infection (MOI) of 10–20 in growth media containing polybrene in an 8 μg/mL final concentration. After 48 h infection, puromycin (2 μg/mL) was added into the media, and virus-positive cells were selected for 10–14 days.

### Immunoblotting

Protein samples were extracted by lysing the transfected cells in 1× Radio immunoprecipitation assay (RIPA) buffer (Beyotime, Shanghai, China) plus proteinase and phosphatase inhibitors (Seivicebio, Wuhan, China) before electrophoresis through sodium dodecyl sulfate-polyacrylamide gel electrophoresis (SDS-PAGE) gels. The resulting gels were electroblotted onto nitrocellulose membranes (Millpore, Shanghai, China), and probing was conducted using antibodies specific for Flag (1:10,000 dilution, Cat No.: 3064, Daian, Wuhan, China), CREB1 (1:2000 dilution, Cat#12208-1-AP, Proteintech), phosphorylated CREB1 (P-CREB1, 1:1000 dilution, Cat#GB114322, Servicebio, Wuhan, China), RSK2 (1:3000 dilution, Cat#23762-1-AP, Proteintech), and β-actin (1:10000 dilution, Cat#81115-1-RR, Proteintech). The goat anti-rabbit IgG conjugated by horseradish peroxidase (1:5000 dilution, Cat#SA00001-2, Proteintech) was employed as the secondary antibody. Protein signals were developed using the enhanced chemiluminescence method (Beyotime). The β-actin served as a loading buffer for normalization.

### Cell counting Kit-8 (CCK-8) cell viability assay

For viability assays, the CCK-8 Kit was applied as described by the manufacturer (Beyotime). Briefly, human MNNG-HOS and U2OS cells after transfections were plated at 3 × 10^3^ cells per well in 96-well culture dishes. After 48 h culture, CCK-8 reagent was used in per well. Cell viability was evaluated by gauging the optical density at 450 nm.

### 5-ethynyl-2’-deoxyuridine (EdU) cell proliferation assay

For proliferation assays, the Click-iT EdU-555 Cell Proliferation Kit (Servicebio) was used as per the accompanying protocols. Briefly, human MNNG-HOS and U2OS cells after transfections (1.5 × 10^4^ cells/well) were maintained in 24-well culture dishes for 48 h. EdU reagent (10 μM) was administrated to culture media for 2 h to label proliferating cells. After being washed by PBS and fixed in 4% paraformaldehyde, the cells were incubated with iF555 working solution for 30 min. Subsequently, the Hoechst 33342 solution was added into each well for nuclear staining. Using fluorescence microscopy (Olympus, Beijing, China), the EdU positive cells (red) were scored relative to total nuclei (blue).

### Transwell migration assay

For migration assays, human transfected MNNG-HOS and U2OS cells resuspending in serum-free media were seeded at 2 × 10^4^ cells per well into 24-transwell inserts. The inserts were then placed into compete growth media, and cell migration was allowed for 24 h. The cells that had migrated to the basal side of the membrane were quantified under an inverted microscope at 100× magnification after crystal violet (0.1%, Servicebio) staining.

### Immunofluorescence

Human MNNG-HOS and U2OS cells after transfections were maintained at 37°C. After being fixed in 4% paraformaldehyde, permeabilized with 0.5% Triton X-100 (Servicebio), and blocked in 3% bovine serum albumin solution (BSA, Beyotime), the cells were probed with primary antibodies including anti-matrix metallopeptidase 9 (anti-MMP9, 1:1000 dilution, Cat#GB11132, Servicebio), anti-proliferating cell nuclear antigen (anti-PCNA, 1:500 dilution, Cat#GB11010, Servicebio), and anti-CREB1 (1:200 dilution, Cat#12208-1-AP, Proteintech). Following the incubation with goat anti-rabbit IgG secondary antibody labeled by Alexa Fluor 488 (1:500 dilution, Cat#GB25303, Servicebio) or Cy3 (1:300 dilution, Cat#GB21303, Servicebio), the cells were further incubated with 4’,6-diamidino-2-phenylindole (DAPI, Servicebio) for nuclear staining. Images were acquired on the Olympus fluorescence microscope, and the fluorescence intensity was analyzed ImageJ (National Institutes of Health, Bethesda, MA, USA).

### Mouse xenograft experiments

After approval by the Institutional Animal Care and Use Committee of the First Affiliated Hospital of Zhengzhou University, ten BALB/c female athymic nude mice age-matched between 5–7 weeks (GemPharmatech, Jiangsu, China) were grown in specific-pathogen-free conditions and mouse xenograft studies were performed. For xenograft experiments, 5 × 10^6^ human U2OS cells transduced with lentiviral particles (vec or LINC00665_18aa) were subcutaneously injected into the rump flanks of nude mice. After 35 days, mice were euthanized by gradually increasing the concentration of CO_2_ in the anesthesia chamber. Xenograft tumors were collected after mice were confirmed to have completely stopped breathing and heartbeat. Each group included five mice. Sections (5 μm) of paraffin-embedded tumors were processed for immunohistochemistry (IHC) as described before [24] using a primary antibody anti-MMP9 (1:1000 dilution, Cat#GB11132, Servicebio), the anti-rabbit secondary antibody (1:300 dilution, Cat#GB23303, Servicebio) and the DAB Assay Kit (Beyotime). All methods were performed in accordance with the relevant guidelines and regulations, and ARRIVE guidelines.

### Immunoprecipitation (IP) and Co-Immunoprecipitation (CoIP)

For IP and CoIP assays, cell extracts were prepared from U2OS cells transfected with LINC00665_18aa expression plasmid or vec control using RIPA buffer plus proteinase and phosphatase inhibitors. Total extractions were co-incubated with protein A/G agarose (Daian, Wuhan, China) and relevant antibody including anti-Flag (1 μg, Cat#80010-1-RR, Proteintech), anti-CREB1 (1 μg, Cat#12208-1-AP, Proteintech), or isotype anti-IgG antibody (1 μg, Cat#30000-0-AP, Proteintech) overnight at 4°C. Beads were collected, and the co-precipitated proteins were harvested for qualitative proteome profiling analysis or immunoblotting.

### Qualitative proteome profiling analysis

The qualitative proteome analysis of the precipitated proteins in IP experiments was performed by Qinglianbio Biotechnology Co., Ltd. (Beijing, China) using RIGOL L-3000 HPLC System (RIGOL, Beijing, China) with Proteome Discoverer2.4 software.

### Dual-luciferase reporter assay

The effect of LINC00665_18aa on CREB1 transcriptional activity was evaluated by luciferase assays by co-transfecting the CREB1-Luc luciferase reporter plasmid, pRL-TK *Renilla* control plasmid (for normalization), and LINC00665_18aa expression plasmid or vec control into U2OS cells. The cells were collected 24 h post-transfection and assayed for luciferase activity using Dual-Luciferase Assay Kit (MedChemExpress, Shanghai, China).

### Statistical analysis

Unless otherwise indicated, mean ± SD values from at least 3 independent replicates were reported in the graphs. For pairwise comparisons, we utilized a two-tailed Student’s *t*-test. For three or more matched groups, we used analysis of variance (ANOVA) followed by a *post hoc* Tukey’s test. Significance for all experiments, evaluated by calculating *P* value, was <0.05.

## Results

### Screen and identification of LINC00665_18aa

To explore the implication of lncRNA-encoded small peptides in OS development, we firstly utilized the Ribo-Seq dataset of human OS cell line U2OS to obtain the lncRNAs associated with ribosomes (count > 0) and selected the longest transcripts. Secondly, using UniProt database (https://www.uniprot.org/) to predict the putative proteins encoded by these lncRNA transcripts, we excluded the peptides matched with human proteome and selected the unmatched peptides (S1 Table). Finally, by combining these unmatched peptides and the lncRNAs that have been identified to have encoding potential in FuncPEP database (https://bioinformatics.mdanderson.org/Supplements/FuncPEP/database.html), a total of seven putative short peptides were obtained (S2 Table). To confirm the finding, we cloned the corresponding sORFs into the Flag tag fusion pcDNA plasmid and evaluated their expression in human U2OS OS cells. Through an immunoblotting method using anti-Flag antibody, three peptides (CRNDE_35aa, CRNDE_71aa and LINC00665_18aa) were validated to express in U2OS cells (Fig 1A). Interestingly, CCK-8 assays revealed that only short peptide LINC00665_18aa encoded by ORF LINC00665_25 significantly suppressed the viability of human MNNG-HOS and U2OS OS cells (Fig 1B). We therefore focused on LINC00665_18aa in this study.

To examine if the start codon of the LINC00665_18aa ORF is active, we constructed two expression plasmids (LINC00665_18aa wt-GFP and LINC00665_18aa mut-GFP) in which the GFP ORF was fused to the C-terminus of the LINC00665_18aa ORF (Fig 1C) and transfected them into human MNNG-HOS and U2OS OS cells. As illustrated in Fig 1D, the LINC00665_18aa-GFP fusion protein was detected in LINC00665_18aa wt-GFP-transfected OS cells. The mutation (LINC00665_18aa mut-GFP), in which the start codon ATG of the LINC00665_18aa ORF was mutated to ATT (Fig 1C), abolished the expression of the LINC00665_18aa-GFP fusion protein (Fig 1D). To eliminate the influence of the GFP tag on protein phenotype, we generated two constructs (LINC00665_18aa wt-Flag and LINC00665_18aa mut-Flag) in which three Flag tags were fused to the C-terminus of the LINC00665_18aa ORF. Consistent with the results of the GFP fusion protein, the LINC00665_18aa-Flag fusion protein was observed in LINC00665_18aa wt-Flag-transfected cells, but not LINC00665_18aa mut-Flag-introduced cells (Fig 1E). Thus, we used the wild-type Flag tag fusion plasmid LINC00665_18aa wt-Flag in the subsequent experiments.

### LINC00665_18aa suppresses OS cell proliferation and migration *in vitro*

**Fig 2:**
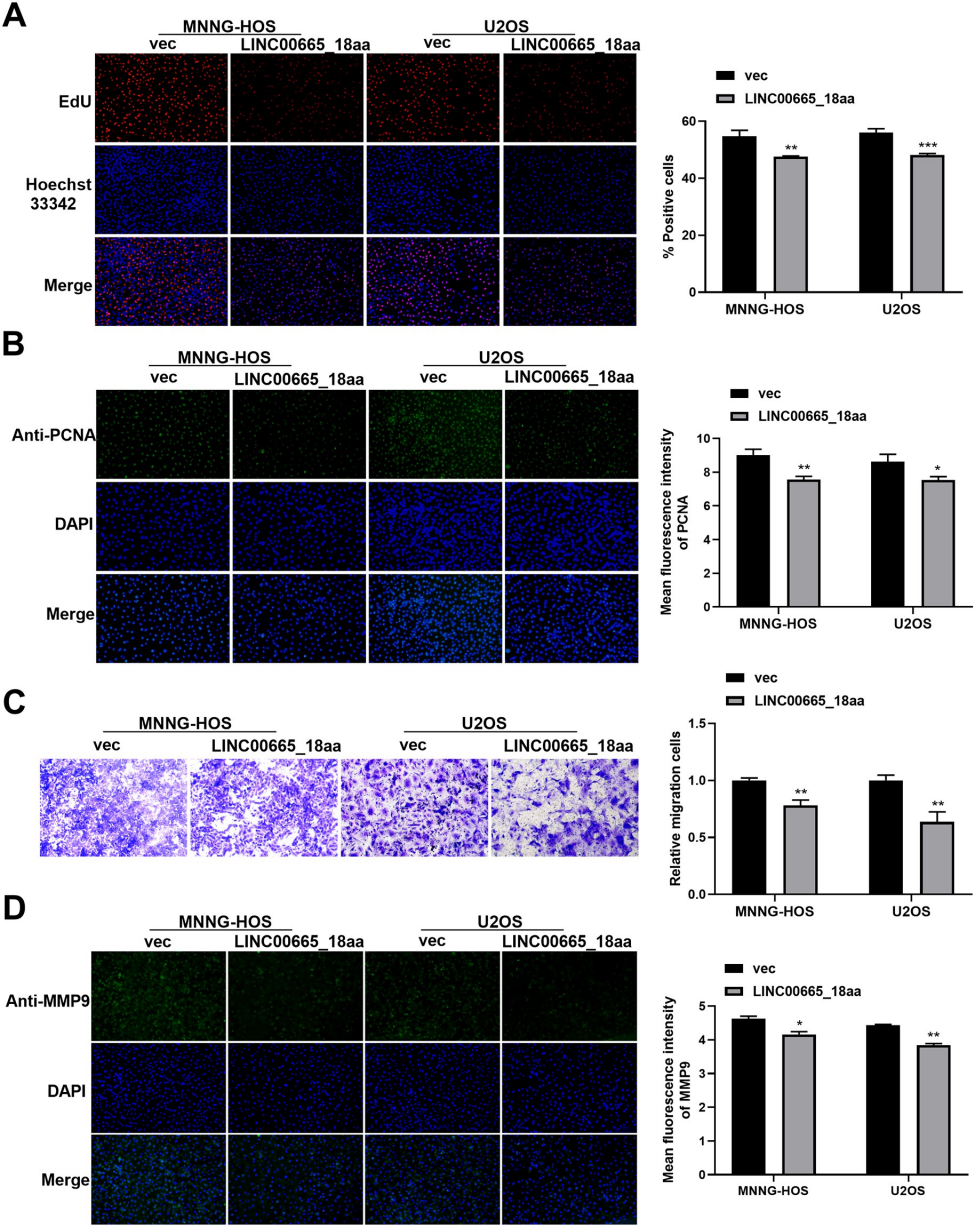
Elevated expression of LINC00665_18aa hinders the proliferation and migration of MNNG-HOS and U2OS cells *in vitro*. (A-D) MNNG-HOS and U2OS OS cells were introduced with constructs expressing LINC00665_18aa or vec controls. (A) Representative images showing a cell proliferation assay performed by EdU assay with transfected cells. (B) PCNA expression in transfected cells by using an anti-PCNA antibody and measuring its fluorescence intensity. (C) Representative transwell pictures depicting a cell migration assay. (D) Representative fluorescence images of MMP9 in transfected cells using an anti-MMP9 antibody. *P<0.05, **P<0.01, ***P<0.001.

Having demonstrated the suppressive effect of LINC00665_18aa on cell viability, we further examined its influence on cell proliferation and migration. The LINC00665_18aa wt-Flag plasmid was used to elevate LINC00665_18aa expression in human MNNG-HOS and U2OS OS cells. Elevated expression of LINC00665_18aa impeded cell proliferation compared with the control group, as evidenced by the decrease of the number of the EdU positive cells (Fig 2A). Moreover, LINC00665_18aa elevation markedly reduced the expression of proliferating marker PCNA (Fig 2B), confirming the inhibitory effect of LINC00665_18aa on cell proliferation. We then used transwell assays to evaluate the effect on cell migration. Human MNNG-HOS and U2OS OS cells expressing LINC00665_18aa exhibited suppressed motility rates compared with the controls (Fig 2C). Additionally, elevated LINC00665_18aa expression reduced the level of migration-related protein MMP9 (Fig 2D), demonstrating the repression of LINC00665_18aa on cell migration. The data establish that LINC00665_18aa exerts inhibitory functions on OS cell proliferation and migration *in vitro*.

### LINC00665_18aa diminishes tumor growth *in vivo*

**Fig 3:**
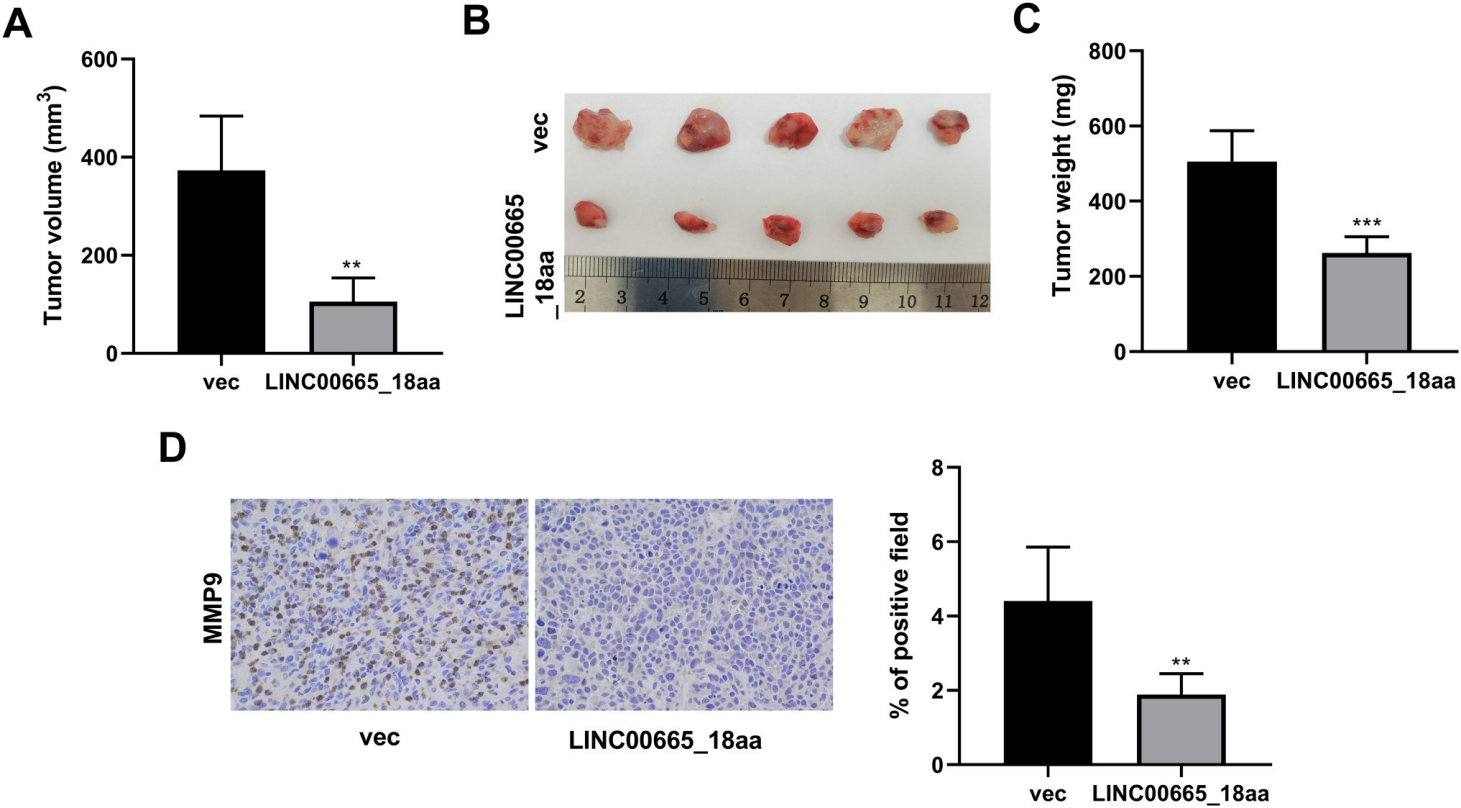
LINC00665_18aa attenuates tumor growth *in vivo*. (A-C) Tumor volume (A), tumor images (B), and the mean weight (C) of xenograft tumors at day 35 (n = 5 per group) derived from subcutaneous injection into the BALB/c nude mice of U2OS cells transduced with lentivirus expressing LINC00665_18aa or a mock control. (D) Representative pictures showing MMP9 staining of sections from LINC00665_18aa lentivirus or vec control xenografts at day 35. **P<0.01, ***P<0.001.

To elucidate whether LINC00665_18aa possesses tumor-inhibitory activity *in vivo*, we conducted xenograft tumor experiments: human U2OS OS cells transduced with lentivirus expressing LINC00665_18aa or a mock control were implanted into BALB/c nude mice by subcutaneous injection. LINC00665_18aa lentivirus-transduced U2OS cells produced remarkably smaller tumors than the same cells transduced with the vec control (Fig 3A–3C). IHC examination of tumor sections showed that LINC00665_18aa expressing tumors had markedly fewer cells stained for MMP9 staining than the controls (Fig 3D). These findings confirm our hypothesis that LINC00665_18aa can impede tumor growth *in vivo*.

### LINC00665_18aa inactivates CREB1, a transcription factor

**Fig 4:**
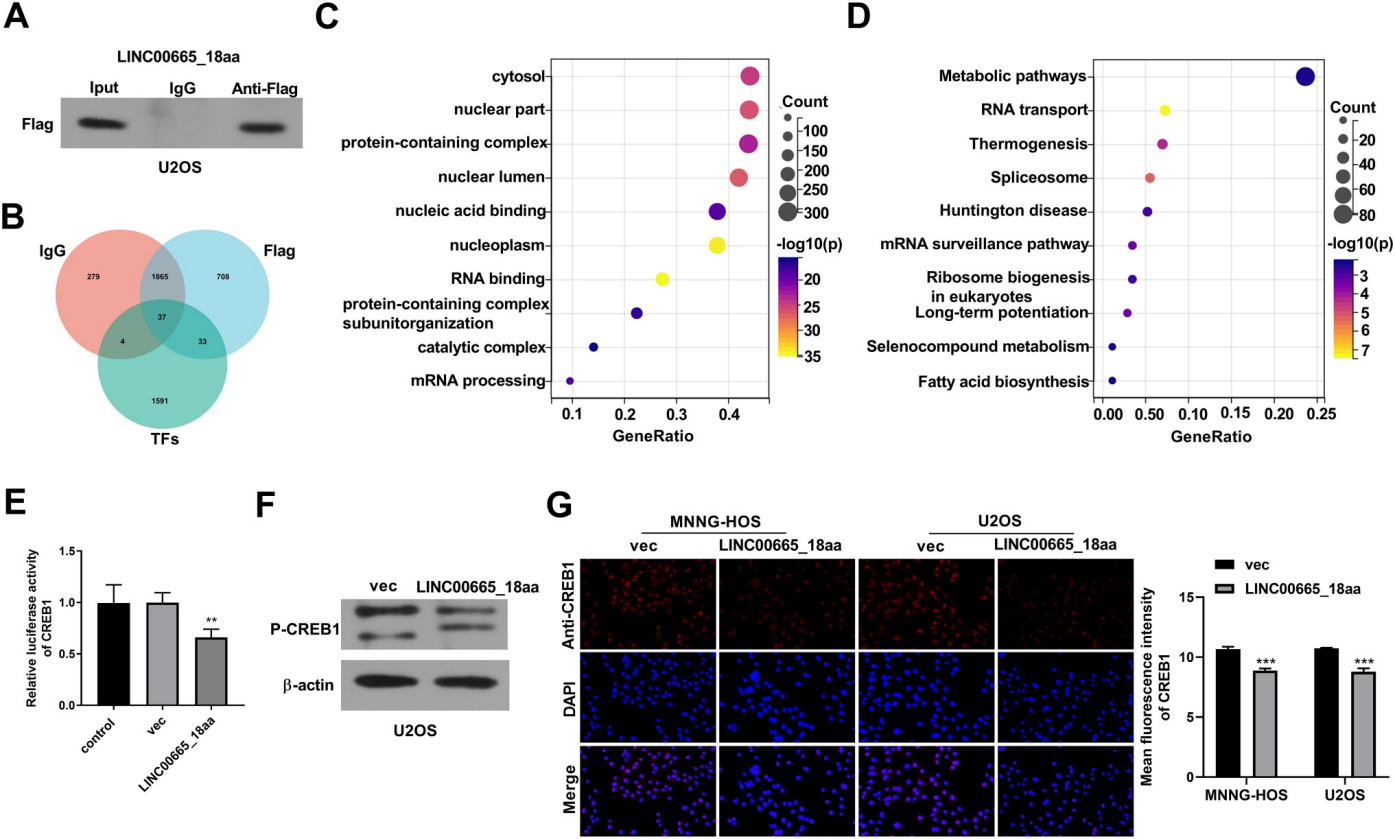
LINC00665_18aa represses the activity of CREB1 in OS cells. (A) IP experiments using an anti-Flag antibody with total extractions of U2OS cells transfected with LINC00665_18aa expression plasmid. Also, immunoblotting analysis of the precipitated proteins in IP experiments using an anti-Flag or anti-IgG antibody. (B) Venn diagram showing the 33 transcriptional factors associated with LINC00665_18aa in U2OS cells. (C and D) The bubble plot showing the top 10 enriched cellular components (C) and the top 10 most enriched KEGG pathways (D) of the precipitated proteins in IP experiments. (E) U2OS cells were co-transfected with the CREB1-Luc luciferase reporter plasmid, pRL-TK *Renilla* control plasmid and LINC00665_18aa expression plasmid or vec control, followed by the measurement of the luciferase activity. (F) Immunoblotting of P-CREB1 in U2OS cells after transfection by LINC00665_18aa expression plasmid or vec control. (G) Representative fluorescence images of CREB1 in the nucleus of MNNG-HOS and U2OS cells after transfection by LINC00665_18aa expression plasmid or vec control using an anti-CREB1 antibody. *P<0.05, **P<0.01, ***P<0.001.

To identify the mechanism by which LINC00665_18aa exerts tumor-inhibitory functions in OS, we performed qualitative proteome profiling analysis after IP experiments: firstly, human U2OS OS cells expressing LINC00665_18aa were lysed and incubated with the anti-Flag antibody, followed by the acquisition of the precipitated proteins from the immunoprecipitates; secondly, the precipitated proteins were conducted with qualitative proteome profiling analysis using HPLC-MS/MS methods. Because LINC00665_18aa expression plasmid was constructed by using the Flag tag fusion plasmid, the existence of LINC00665_18aa in the precipitated proteins was confirmed by immunoblotting with anti-Flag antibody (Fig 4A). Using the qualitative proteome analysis of isotype IgG antibody to rule out protein impurity, a total of 741 unique proteins were pulled down in the anti-Flag antibody group (Fig 4B, S3 Table). We then performed GO enrichment analysis and KEGG pathway enrichment analysis of the 741 unique proteins using DAVID database. Results showed that the 741 precipitated proteins were closely associated with RNA binding (Fig 4C), and the RNA transport pathway was predicted as the most prominent enriched pathway (Fig 4D). By combining the 1665 all human transcriptional factors from JASPAR database (https://jaspar.genereg.net/) and the 741 proteins, a total of 33 transcriptional factors were found to be associated with LINC00665_18aa (Fig 4B, S4 Table).

CREB1 is a critical transcriptional factor that exerts an important function in normal development and human disease [16, 25]. More importantly, CREB1 operates as a potent tumor driver in OS [18, 19, 26]. We therefore focused on CREB1 in this study and hypothesized that LINC00665_18aa might regulate CREB1 activation in OS cells. To confirm this, we evaluated whether LINC00665_18aa impacts the transcriptional activity, nuclear localization, and phosphorylation of CREB1. Luciferase assays revealed that increased expression of LINC00665_18aa upon construct transfection led to a strong repression of luciferase activity of the CREB1-Luc luciferase reporter plasmid, demonstrating that LINC00665_18aa repressed the transcriptional activity of CREB1 (Fig 4E). Immunoblotting results showed that increased expression of LINC00665_18aa reduced P-CREB1 level in human U2OS OS cells compared with the control group, indicating that LINC00665_18aa weakened the phosphorylation of CREB1 (Fig 4F). Furthermore, LINC00665_18aa hindered the nuclear localization of CREB1, as evidenced by the reduction of CREB1 fluorescence intensity in the nucleus of human MNNG-HOS and U2OS OS cells (Fig 4G). All these findings together support the notion that LINC00665_18aa targets CREB1 and represses its activity.

### LINC00665_18aa weakens the interaction of CREB1 and RSK2

**Fig 5:**
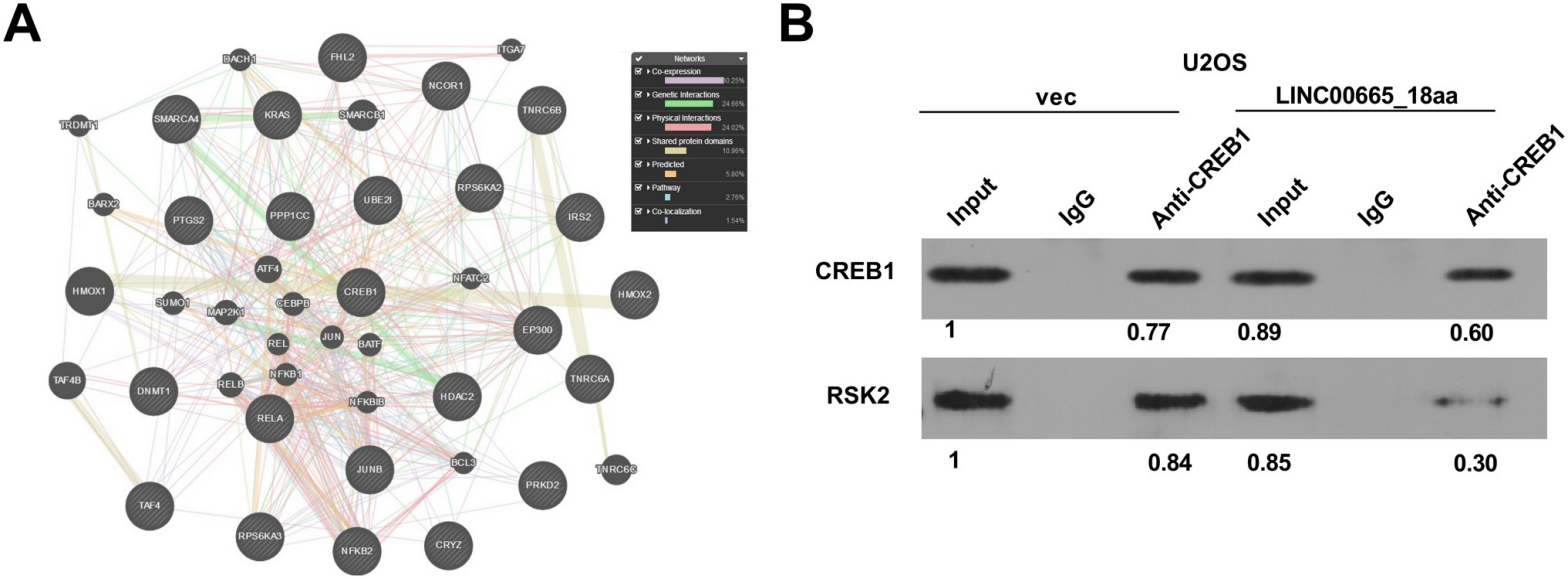
LINC00665_18aa diminishes the interaction of CREB1 and RSK2. (A) Schematic diagram showing a part of interaction network of these precipitated proteins pulled down by LINC00665_18aa. (B) U2OS cells transfected with LINC00665_18aa expression plasmid or vec control were lysed and incubated with an anti-CREB1 antibody. The precipitated proteins were detected by immunoblotting using an anti-CREB1 or anti-RSK2 antibody.

Further, we analyzed the interaction network of the 741 precipitated proteins pulled down by LINC00665_18aa using String database (https://cn.string-db.org/). Intriguingly, we found that CREB1 can interact with 22 proteins (Fig 5A, S5 Table). Using Genemania tool (http://genemania.org/) to analyze the interaction relationships between CREB1 and the 22 proteins, we found that CREB1 and RSK2 have a variety of interaction relationships, such as co-localization and co-expression (Fig 5A). CREB1 can associate with RSK2 and thus modulates several important signaling pathways [27] and plays a significant role in OS development [23]. To explore whether LINC00665_18aa can influence the interaction between CREB1 and RSK2, we performed CoIP experiments: human U2OS OS cells transfected with LINC00665_18aa expression plasmid or vec control were lysed and incubated with an anti-CREB1 antibody, followed by the detection of the precipitated proteins by immunoblotting. Compared with the vec control (gray value of RSK2: CREB1 = 1.09), LINC00665_18aa impaired the interaction of CREB1 and RSK2 (gray value of RSK2: CREB1 = 0.52) (Fig 5B).

### Elevated expression of CREB1 reverses the inhibitory effects of LINC00665_18aa on OS cell proliferation and migration

**Fig 6:**
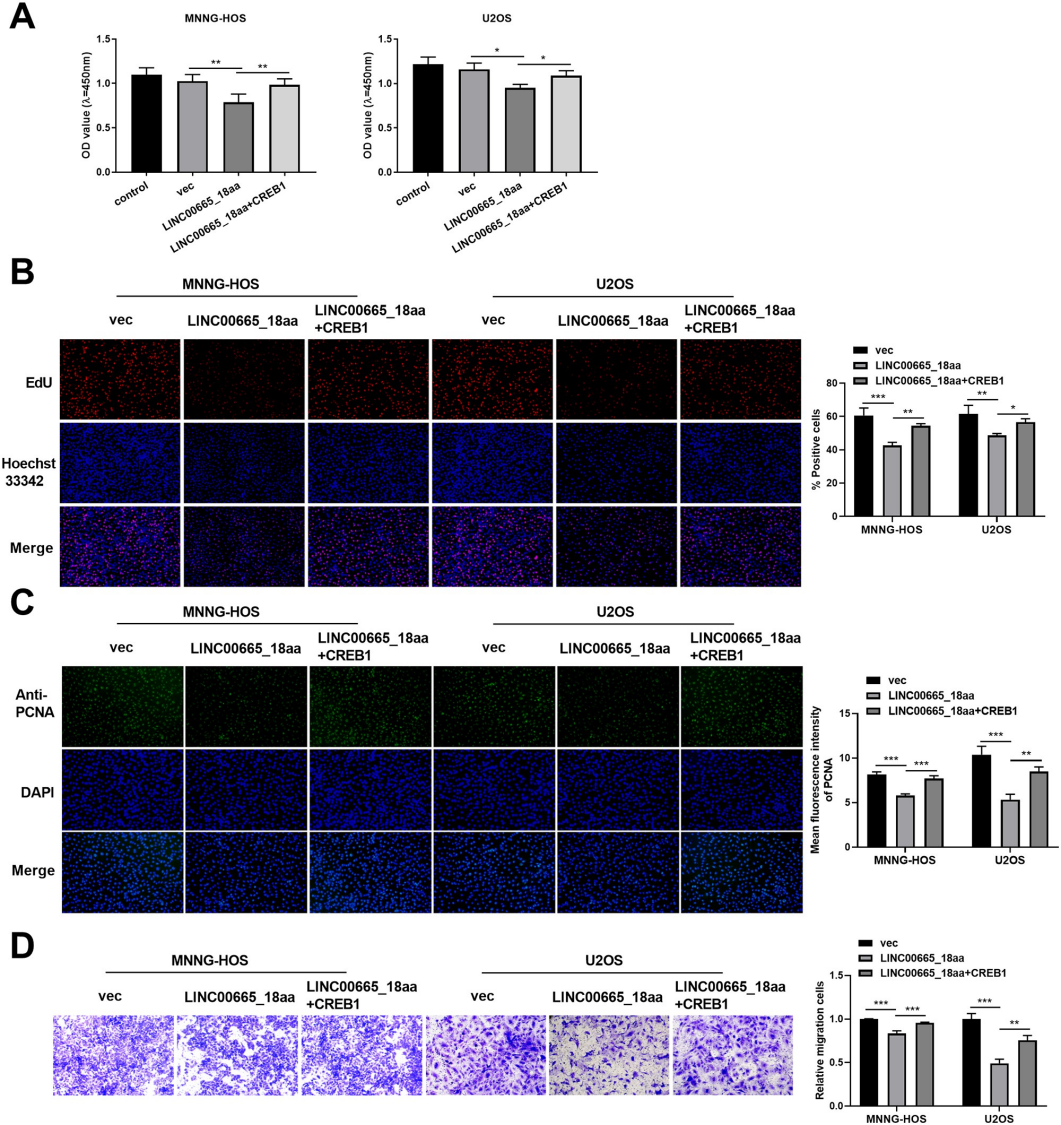
LINC00665_18aa-mediated regulation of CREB1 impacts cell proliferation and migration. (A-D) MNNG-HOS and U2OS OS cells were introduced with or without vec control, LINC00665_18aa expression plasmid, or constructs expressing LINC00665_18aa and CREB1. (A) Cell viability by CCK-8 assay performed with cells transfected as indicated. (B) Representative images showing a cell proliferation assay performed by EdU assay with cells transfected as indicated. (C) Representative fluorescence images of PCNA in transfected cells using an anti-PCNA antibody. (D) Representative transwell pictures depicting a cell migration assay performed with cells transfected as indicated. *P<0.05, **P<0.01, ***P<0.001.

To directly determine whether the inhibitory effects of LINC00665_18aa are due to the inactivation of CREB1, we performed a rescue experiment by introducing CREB1 expression construct into LINC00665_18aa-expressing human OS cells. Increased expression of CREB1 in human MNNG-HOS and U2OS OS cells rescued LINC00665_18aa-driven cell viability and proliferation defects (Fig 6A and 6B). Through fluorescence intensity analysis, LINC00665_18aa and CREB1 co-expressing cells showed a clear augmentation of PCNA expression compared with the LINC00665_18aa controls (Fig 6C). Moreover, CREB1 increase significantly abrogated LINC00665_18aa-imposed suppression of migration of human MNNG-HOS and U2OS OS cells (Fig 6D). Together, these results suggest that LINC00665_18aa exerts inhibitory effects on cell proliferation and migration, at least in part, through CREB1.

## Discussion

Although lncRNAs were initially defined as ncRNAs, a number of lncRNAs have recently been demonstrated to actually associate with ribosomes and contain sORFs that can be translated into short peptides [28]. The demonstration of functionally lncRNA-encoded short peptides in cancer biology suggests that these lncRNAs might possess dual roles, with both RNA and peptides, in carcinogenesis [9]. For example, a small peptide ASRPS with 60-aa encoded by LINC00908 functions as a potent anti-tumor polypeptide in triple-negative breast cancer by modulating STAT3 activity [29]. The LINC00998-encoded short peptide SMIM30 can enhance the tumorigenesis of hepatocellular carcinoma through the regulation of the SRC/YES1/MAPK pathway [30]. Moreover, a short 130-aa peptide SRSP encoded by LOC90024 contributes to colorectal cancer development by promoting the binding of SRSF3 to Sp4 transcription factor [31].

Previous reports have provided evidence that LINC00665, an originally annotated as a lncRNA transcript, operates as a tumor promoter in OS by working as a sponge of certain miRNAs [12, 13]. In the current work, we discover, for the first time, that lncRNA LINC00665 can encode an 18-aa-long short peptide LINC00665_18aa in human OS cells. Contrary to the function of its maternal lncRNA LINC00665, LINC00665_18aa acts as a novel suppressor of OS cell proliferation and motility *in vitro* and tumor growth *in vivo*. These above results are also supported by the expression alteration of PCNA and MMP9 in OS cells because PCNA is an essential factor in DNA metabolism and replication and a well-known cell proliferation marker [32], and MMP9 expression is closely associated with OS cell metastasis [33]. Consistent with our findings, although lncRNA LINC00665 has been reported to exert a promoting effect on the progression of breast cancer [34, 35], its 52-aa peptide CIP2A-BP functions as an anti-tumor factor in TNBC via the PP2A/PI3K/AKT/NF-κB pathway by binding the oncogene CIP2A [14]. However, Li *et al*. found that both lncRNA LINC00665 and its short peptide CIP2A-BP promoted HCC development through the enhancement of cancer cell growth and motility [15]. These data suggest that lncRNA LINC00665 can encode different short peptides in different types of cancer cells, and the effect of short peptides may be inconsistent with that of the maternal lncRNAs.

Using qualitative proteome analysis after IP experiments with anti-Flag antibody, we found many proteins bound to LINC00665_18aa and selected transcription factor CREB1 for further exploration in this study. Numerous studies have established the promoting role of CREB1 in the carcinogenesis of multiple cancers, such as liver cancer, colorectal cancer, and prostate cancer [36–38]. In OS, activation of CREB1 is crucial for cancer initiation and maintenance [26]. Moreover, lncRNA ELFN1-AS1 and UCA1 up-regulate CREB1 expression to contribute to OS progression by sponging certain miRNAs [18, 19]. However, no studies proved whether CREB1 can be regulated by lncRNA-encoded peptides. In the current study, LINC00665_18aa is demonstrated to suppress the activation of CREB1 in human U2OS cells. Furthermore, our findings confirm that LINC00665_18aa exerts inhibitory effects on OS cell proliferation and migration, partly through CREB1.

RSK2 is a strong oncogene in OS [20, 21] and can activate CREB1 [22]. The association and interaction between RSK2 and CREB1 can modulate several signaling pathways, including the toll and NF-κB pathways, in multiple myeloma cells [27]. Moreover, the activation of the RSK2/CREB1 pathway is associated with multiple myeloma development by affecting cancer cell growth and survival [39]. Additionally, the RSK2/CREB signaling pathway can promote the progression of various cancers [40], including OS [23]. In the current report, bioinformatics data showed the co-localization and co-expression of CREB1 and RSK2. Importantly, CoIP experiments validated that LINC00665_18aa impairs the interaction between CREB1 and RSK2 in OS cells.

With these findings, we envision that the lncRNA-encoded short peptide LINC00665_18aa has the potential to develop novel therapies against OS. However, there are still several limitations in the current research. When we predicted the lncRNAs with encoding potential in U2OS OS cells, we selected their longest transcripts. The selection method is limited and may miss other transcripts with sORFs and encoding potential. There is no doubt that how to predict the coding potential of lncRNA more effectively is still a challenge for the further investigations. Additionally, the *in vivo* assays revealed the inhibitory impact of LINC00665_18aa on xenograft tumor growth, which is lacking the evidence about the involvement of CREB1/RSK2 interaction in the regulation of LINC00665_18aa. Further *in vivo* studies about the novel mechanism are warranted in future work. Although our study demonstrates the tumor-inhibitory function of LINC00665_18aa, its safety and long-term efficacy in various experimental models should be further determined.

## Conclusion

Our study shows that lncRNA LINC00665 can encode an 18-aa-long short peptide LINC00665_18aa. The short peptide diminishes OS cell proliferation and migration by repressing the transcriptional activity of CERB1 and impairing the interaction between CREB1 and RSK2. These findings broaden the diversity and breadth of lncRNAs in human carcinogenesis and provide a new basis for cancer therapeutics through the functions of the short encoding peptides.

## Supporting information

S1 Table The lncRNA-encoded small peptides unmatched with human proteome in U2OS cells. (XLSX)

S2 Table The information of the seven putative lncRNA-encoded short peptides in U2OS cells. (XLSX)

S3 Table The 741 unique proteins pulled down by LINC00665_18aa in U2OS cells. (XLSX)

S4 Table The 33 transcriptional factors that can be pulled down by LINC00665_18aa in U2OS cells. (XLSX)

S5 Table The 22 proteins interacted with CREB1 using String database. (XLSX)

S1 File Raw images of western blot. (PDF)
