# Supplementary material for: A short peptide LINC00665_18aa encoded by lncRNA LINC00665 suppresses the proliferation and migration of osteosarcoma cells through the regulation of the CREB1/RPS6KA3 interaction
Source: PLoS One. 2023 Jun 7;18(6):e0286422. doi: 10.1371/journal.pone.0286422 (PMC10246827; doi:10.1371/journal.pone.0286422)
Supplement: S1 File — (PDF) [file pone.0286422.s006.pdf]

Figure1A

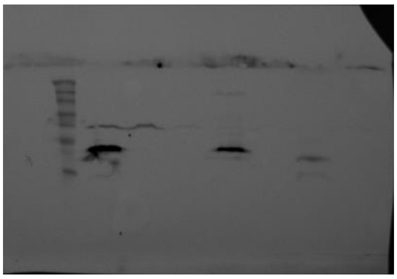

Flag

Figure1E

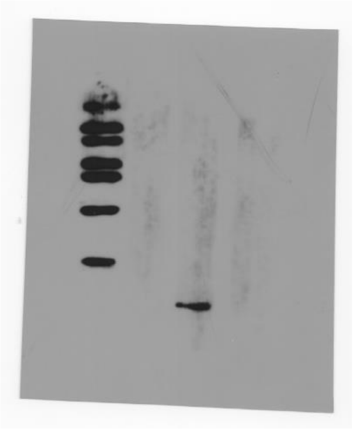

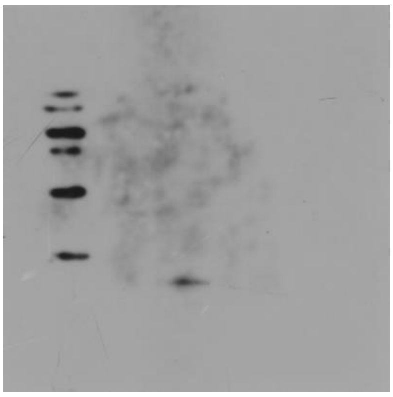

Flag-MNNG-HOS

Flag-U2OS

Figure4A

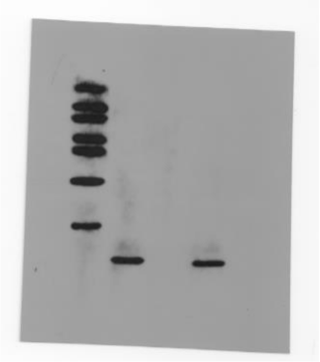

Flag

Figure 4F

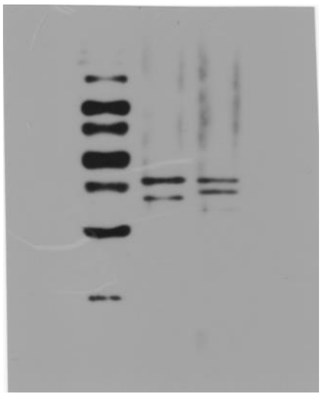

p-CREB1_1

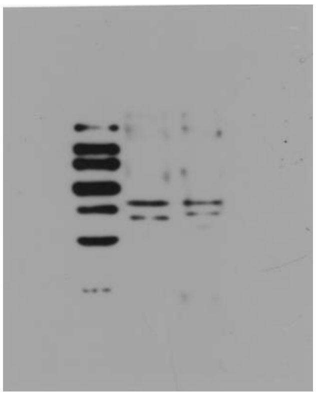

p-CREB1_2

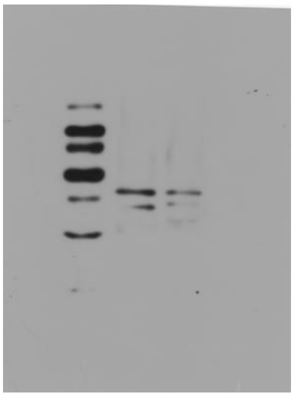

p-CREB1_3

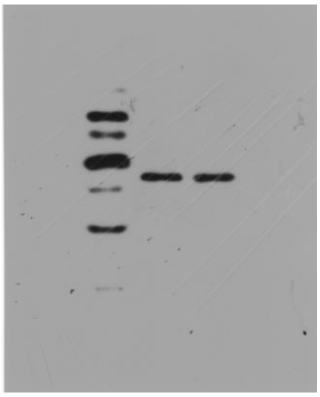

β-actin_1

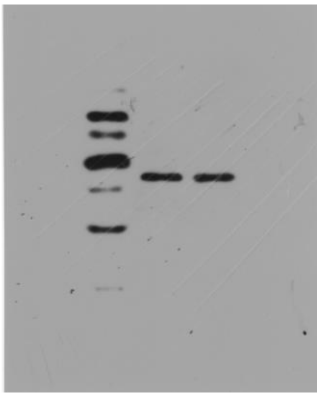

β-actin_2

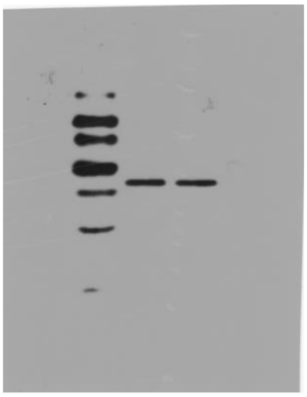

β-actin_3

Figure5B

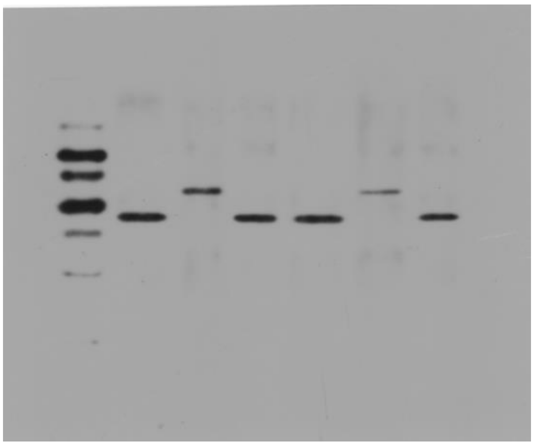

CREB1

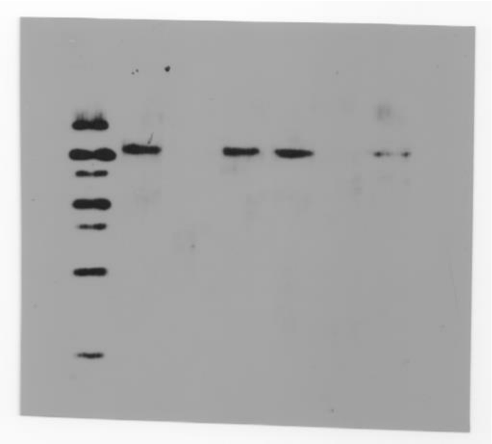

RSK2
